# Local Administration of Caloric Restriction Mimetics to Promote the Immune Control of Lung Metastases

**DOI:** 10.1155/2019/2015892

**Published:** 2019-06-20

**Authors:** Valentino Le Noci, Michele Sommariva, Francesca Bianchi, Tiziana Triulzi, Elda Tagliabue, Andrea Balsari, Lucia Sfondrini

**Affiliations:** ^1^Molecular Targeting Unit, Fondazione IRCCS Istituto Nazionale dei Tumori, Milan 20133, Italy; ^2^Dipartimento di Scienze Biomediche per la Salute, Università degli Studi di Milano, Milan 20133, Italy

## Abstract

Caloric restriction mimetics (CRMs), compounds that mimic the biochemical effects of nutrient deprivation, administered via systemic route promote antitumor effects through the induction of autophagy and the modulation of the immune microenvironment; however, collateral effects due to metabolic changes and the possible weight loss might potentially limit their administration at long term. Here, we investigated in mice local administration of CRMs via aerosol to reduce metastasis implantation in the lung, whose physiologic immunosuppressive status favors tumor growth. Hydroxycitrate, spermidine, and alpha-lipoic acid, CRMs that target different metabolic enzymes, administered by aerosol, strongly reduced implantation of intravenously injected B16 melanoma cells without overt signs of toxicity, such as weight loss and changes in lung structure. Cytofluorimetric analysis of lung immune infiltrates revealed a significant increase of alveolar macrophages and CD103+ dendritic cells in mice treated with CRMs that paralleled an increased recruitment and activation of both CD3 T lymphocytes and NK cells. These effects were associated with the upregulation of genes related to M1 phenotype, as IL-12 and STAT-1, and to the decrease of M2 genes, as IL-10 and STAT-6, in adherent fraction of lung immune infiltrate, as revealed by real-time PCR analysis. Thus, in this proof-of-principle study, we highlight the antitumor effect of CRM aerosol delivery as a new and noninvasive therapeutic approach to locally modulate immunosurveillance at the tumor site in the lung.

## 1. Introduction

The study of cancer metabolism is now receiving substantial attention for its implications in the biology of cancer and the possibility to find new therapeutic interventions. Recently, caloric restriction mimetics (CRMs), compounds mimicking the biochemical effects of nutrient deprivation, have revealed antitumor properties [1]. There are several examples of natural molecules able to target different metabolic enzymes. For instance, hydroxycitrate and alpha-lipoic acid induce cytosolic AcCoA depletion, spermidine or curcumin inhibits acetyltransferase activity, and resveratrol promotes histone deacetylation [2, 3]. Targeting cancer metabolism using different combinations of these agents, administered systemically or by drinking water, has been demonstrated to decrease tumor cell growth in different mouse models [4–6]. Moreover, treatment with CRMs, as hydroxycitrate and spermidine, has been reported to improve chemotherapy efficacy [7].

The biological activity of these compounds mostly relies on their ability to induce autophagy, shaping the tumor microenvironment (TME), and promote anticancer immunosurveillance [7]. Indeed, autophagy induces the release of adenosine triphosphate (ATP) in the extracellular space, acting as a danger signal, and attracts antigen-presenting cells (APCs) in the tumor bed, resulting in the activation of an adaptive immune response against tumors. On the other hand, autophagy has been known to prevent the upregulation of CD39 on tumor cells, an ecto-ATPase that converts extracellular immunostimulatory ATP into immunosuppressive adenosine diphosphate (ADP) and that recruits regulatory T cells (Tregs) in the TME [8], and we also already highlighted that tumor cells are able to finely tune the ATP/ADP levels [9]. Thus, pharmacologically induced autophagy may represent a novel strategy to reduce immunosuppression and, at the same time, enhance the immune response against cancer. However, although some autophagy-modulating drugs have been already tested in early phase 1 clinical trial [10], major concerns remain because of their possible toxic side effects when systemically administered.

Since lungs are constantly exposed to inhaled antigens, these organs represent a particularly immunosuppressive milieu to limit excessive immune response. It has been speculated that cancer cells can harness this unique environment for their implantation and growth, explaining, at least in part, the high incidence of lung metastases arising from several types of tumors [11].

Aerosolization is an efficient and noninvasive method of delivering molecules to the lung in order to improve local tissue concentration, limiting potential adverse effects induced by a systemic administration, and we previously demonstrated its usefulness to modulate lung microenvironment [12, 13].

In the present study, we evaluated local administration by aerosol delivery of hydroxycitrate, spermidine, and alpha-lipoic acid to reduce tumor implantation in the lung of mice intravenously injected with B16 melanoma cells, as a new therapeutic approach to locally modulate antitumor immune response.

## 2. Material and Methods

### 2.1. Cell Lines and Reagents

B16 mouse melanoma cells (American Type Culture Collection (ATCC), Rockville, MD, USA) and N202.1A cells, derived from a spontaneous mammary carcinoma in an FVB-neuN transgenic mouse [14], were routinely maintained at 37°C in a 5% CO2 atmosphere in RPMI 1640 medium and Dulbecco's modified Eagle's medium (DMEM) (Thermo Fisher Scientific Inc., Waltham, MA, USA), respectively, supplemented with 10% fetal bovine serum (Thermo Fisher Scientific) and 2 mM glutamine (Sigma-Aldrich, St. Louis, MO, USA).

Cell lines were authenticated by the Fragment Analysis Facility at Fondazione IRCCS Istituto Nazionale dei Tumori (Milan, Italy) using the GenePrint 10 System (Promega, Madison, WI, USA), and cultures were regularly tested for Mycoplasma by using the MycoAlert Plus Kit (Lonza Group Ltd., Basel, Switzerland). Hydroxycitrate, spermidine, and alpha-lipoic acid were purchased from Sigma-Aldrich. ^51^Cr (1 mCi) was purchased from PerkinElmer (Waltham, MA, USA, NEZ030S001MC).

### 2.2. Mice and Experimental Protocols

Female C57BL/6 and FVB mice, aged 6-8 weeks (Charles River Laboratories, Calco, Italy), were maintained in laminar flow rooms at constant temperature and humidity, with food and water given ad libitum. Mice were treated with alpha-lipoic acid (10 mg/kg), spermidine (5 mg/kg) (5 days/week at 12 h intervals), and hydroxycitrate (14 mg) (5 days/week) dissolved in 5 ml of saline starting 1 day after the intravenous (i.v.) injection of 3 × 10^5^ N202.1A carcinoma cells or 5 × 10^5^ B16 melanoma cells, respectively, and continuing throughout the experiment. Aerosolization was performed using a tower inhalation system (IES 306 Inhalation Towers, EMMS, Havant, UK). The suspensions were placed in the nebulizer (Aeroneb Lab Micropump Nebulizer, EMMS) and used to treat groups of 6 mice by exposure to aerosol for 25 min. In all experiments, mice were weighed and inspected for any sign of sufferance twice weekly and euthanized at day 21 after tumor injection to count macroscopic lung metastases.

The experimental protocols were carried out in accordance with the Italian law D.Lgs. 26/2014, and animal experimentation was performed following the guidelines drawn up by Fondazione IRCCS Istituto Nazionale dei Tumori Institutional Animal Welfare Body according to Workman et al. [15].

### 2.3. Histological, Immunofluorescence, and Immunohistochemical Examination of Lungs

To exclude any effects of aerosolized CRM molecules on the architecture and structure of the lung parenchyma, lung samples were analyzed as described [16].

To analyze autophagy induced by CRM aerosolization, immunohistochemical analyses were performed to detect LC3B molecule. IHC was carried out on formalin-fixed paraffin-embedded lung sections using a protocol previously described with slightly modifications [17]. Briefly, sections were deparaffinized and underwent heat-induced epitope retrieval at pH 6 for 10 min at 95°C in citrate buffer. Slides were rinsed and treated with PBS containing BSA 1% for 30 min to reduce nonspecific background staining and then incubated ON at 4°C with anti-LC3B 1 : 450 (Ab 48394 Abcam). Staining was revealed using the Alkaline Phosphatase kit (LEICA Biosystems) according to the manufacturer's protocol. Images were acquired as previously described [18].

### 2.4. Isolation of Lung Suspensions

Isolation of lung immune cells was performed as described [13]. Briefly, lungs were digested in DMEM medium containing collagenase (300 U/ml) and hyaluronidase (100 U/ml) (Stemcell Technologies, 07912) for 1 h at 37°C. Cell suspensions were then filtered through 70 *μ*m cell strainers and, after lysis of red blood cells, were directly stained for flow cytometry or plated to separate adherent and nonadherent cell fractions as described [19].

### 2.5. Flow Cytometry

To analyze immune lung infiltration, lung suspensions were stained as previously described [20] using the following directly conjugated antibodies: CD3e FITC (Miltenyi, Miltenyi Biotec GmbH, Bergisch Gladbach, Germany, clone 145-2C11); CD11b PE (BD Biosciences, San Jose, CA USA, clone M1/70); CD11c PECY7 (Thermo Fisher Scientific-eBiosciences, clone N418); CD45 APCeFluor780 (Thermo Fisher Scientific-eBioscience, clone 30-F11); CD49b PE (Miltenyi, clone DX5); CD69 APC (Miltenyi, clone H1.2F3); CD103 APC (Miltenyi, clone REA789); B220 PERCPVio700 (Miltenyi, clone RA3-6B2); and mPDCA-1 PE (Miltenyi, clone JF05-1C2.4.1). A purified rat anti-mouse CD16/CD32 MAb (Thermo Fisher Scientific-eBioscience, clone 93) was used to block nonspecific binding to mouse Fc receptors. The cells were analyzed using a FACSCanto flow cytometer (BD Biosciences) and FlowJo software (TreeStar). All analyses were performed using gating on CD45+ live cells after doublet exclusion.

### 2.6. Quantitative PCR Analysis

RNA was isolated using QIAzol (QIAGEN) from adherent cells (containing macrophage/myeloid-derived cells) according to the manufacturer's instructions. Reverse transcription was performed using a High-Capacity RNA-to-cDNA Kit (Applied Biosystems-Thermo Fisher Scientific). Real-time PCR was performed using TaqMan® Fast Universal PCR Master Mix (Applied Biosystems-Thermo Fisher Scientific) and SDS 2.4 on a 7900HT Fast Real-Time PCR System (Applied Biosystems-Thermo Fisher Scientific), as we previously described [21]. The following TaqMan® gene expression assays (Applied Biosystems-Thermo Fisher Scientific) were used in real-time PCR analyses: STAT1 (assay ID: Mm01257286_m1), STAT6 (assay ID: Mm01160477_m1), Il10 (assay ID: Mm01288386_m1), Il12 (assay ID: Mm00434169_m1), IL8 (assay ID: Mm04207460_m1), IFN*γ* (assay ID: Mm01168134_m1), and TNF-*α* (assay ID: Mm00443258_m1). The expression of each gene was normalized to *β*2m (assay ID: Mm00437762_m1). PCR data were analyzed using the 2-ΔCt method.

### 2.7. In Vitro Cytotoxicity Assays

The ability of effector immune cells from the lung immune infiltrates of mice to promote antitumor activity was evaluated by measuring cytotoxic activity of nonadherent cells obtained from the lung suspensions on 51Cr-B16 target cells as described [19]. The radioactivity of the supernatant (80 *μ*l) was measured as described [22].

### 2.8. Statistical Analysis

Differences among groups were compared using a two-tailed unpaired Student's *t*-test and considered significant at *p* ≤ 0.05. All analyses were performed using GraphPad Prism version 5.0 for Windows (GraphPad Software).

## 3. Results and Discussion

CRMs, whose antitumor effect has been demonstrated in different preclinical models [23], combine the advantages of caloric restriction (CR) without significantly reducing food intake. As observed during CR, CRMs lead to several physiological changes including reduction in glucose, insulin, and triglyceride blood concentration paralleled by an increase of blood ketone body levels [24]. Therefore, CRM systemic administration can potentially determine several adverse effects on healthy tissues. The possible weight loss observed after long-term CRM treatment might also represent a serious problem, especially for cancer patients at risk of cachexia [10]. Thus, we hypothesized that the local delivery of these drugs directly to the airways by aerosol administration could be a promising strategy to limit their distribution to the systemic circulation [10].

To evaluate whether aerosolized CRMs are able to reach the alveolar space and to control metastasis implantation, female C57BL/6 mice, intravenously (i.v.) injected with murine B16 melanoma cells, were treated with hydroxycitrate, spermidine, and alpha-lipoic acid. B16 cell is a low immunogenic tumor that establishes a highly immunosuppressive microenvironment recruiting tumor-infiltrating macrophages (TAMs) and MDSC [25, 26], when implanted in lungs.

The combination of hydroxycitrate, spermidine, and alpha-lipoic acid was chosen to concomitantly target different metabolic enzymes, based on the complexity of the aerobic glycolytic pathway and on previous published results that demonstrated the superior efficacy of combinations of these compounds than single agents alone in preclinical cancer models [6].

**Figure 1:**
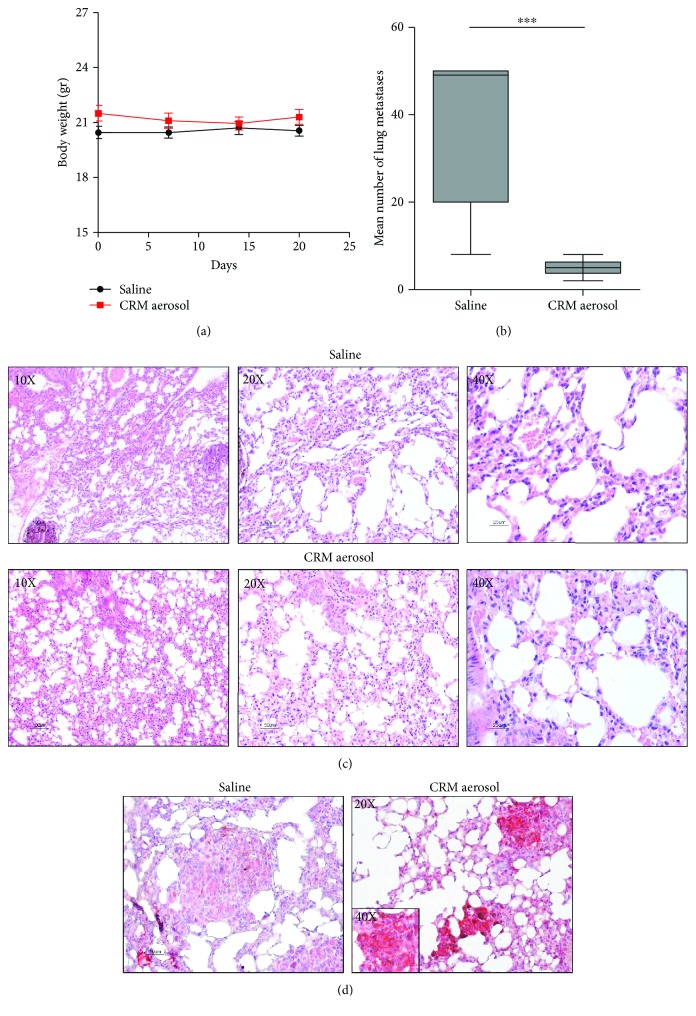
Effects of CRM aerosolization on body weight, on the growth of experimental B16 lung metastases, and on lung histology. Body weights (a) and number of macroscopic lung metastatic foci (b) of mice (9-10 mice/group) treated starting 1 day after i.v. injection of B16 melanoma cells with aerosolized saline or combined CRMs for 3 weeks. Representative images showing histopathological evaluation of hematoxylin- and eosin-stained lung tissue sections (c) and IHC analysis of LC3B staining in tumor nodules (d) (magnification: ×200) of saline and CRM-treated mice. ^∗∗∗^ *p* ≤ 0.001 by Student's *t*-test.

Treatments with aerosolized CRMs were well-tolerated, as indicated by the absence of signs of toxicity, such as hunching, ruffled fur, and difficulty breathing. Moreover, no weight loss was observed in CRM-treated mice (Figure 1(a)). At the end of the experiment, the number of macroscopic melanotic metastases was significantly reduced in CRM-treated mice, as compared to control group (*p* ≤ 0.0001) (Figure 1(b)). Histological analysis of lung samples revealed no alterations in lung parenchyma of mice treated with combined CRMs (Figure 1(c)). LC3-positive staining, the widely used marker for autophagosome [27], was strongly detected by IHC in CRM-treated lungs (Figure 1(d)), confirming that aerosolized CRMs induced autophagy in tumor nodules. The lack of toxicity and the ability to affect lung metastatization indicate that CRM aerosol delivery may represent a novel weapon in cancer treatment.

Inducing autophagy, CRMs have been demonstrated to influence tumor growth by targeting cancer cell metabolism. This activity has been demonstrated to play dual effects as it not only prevents tumor initiation but also promotes tumor progression by assisting in hypoxia-induced switch to anaerobic glycolysis [10]. However, autophagy has been reported to also affect tumor growth by the modulation of the immune microenvironment through the release of immunostimulatory danger signals [28]. Therefore, we evaluated the lung immune contexture after CRM aerosol administration.

**Figure 2:**
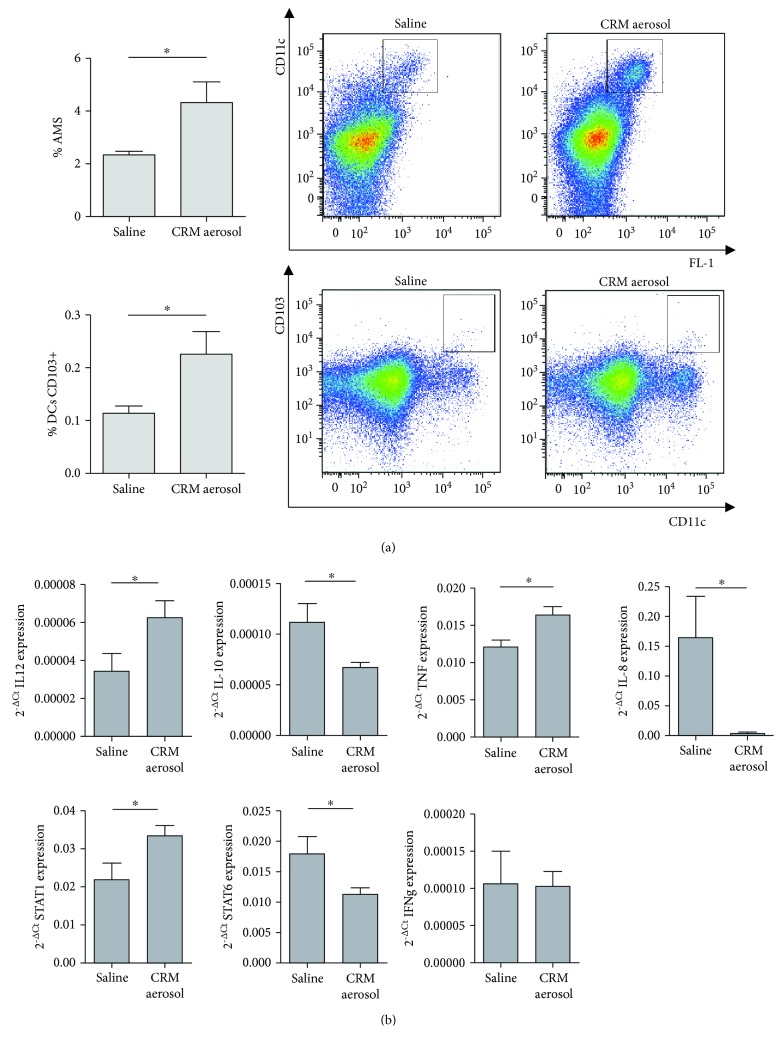
Modification of lung immune contexture induced by CRM aerosolization. Bars (mean ± SEM) and representative dot plots of the percentage of AMs (identified as CD45+/FL-1+CD11c+ cells) and of CD103+ DCs (identified as CD45+/CD11b-/CD103+CD11c+ cells) evaluated in lung suspensions of 4 mice/group injected with B16 melanoma cells and aerosolized with saline or combined CRMs (a). Mean relative expression ± SEM of IL10, IL12, STAT1, STAT6, TNF-*α*, IFN*γ*, and IL-8 mRNA levels, evaluated by real-time PCR in adherent cell fraction of digested lungs (4-8 mice/group) (b). Results are presented as 2^-ΔCt^. ^∗^ *p* ≤ 0.05 by Student's *t*-test.

Cytofluorimetric analysis of immune infiltrate obtained after tumor-bearing lung enzymatic digestion showed a significant increase of alveolar macrophages (AMs) (FL-1+CD11c+ cells) and CD103+ dendritic cells (DCs) (CD11b-CD103+CD11c+ cells) in mice treated with CRM aerosol as compared to control group (Figure 2(a)). No difference was observed in conventional CD11b+CD11c+ DCs (data not shown). CD103+ DCs represent the major DC population in the lung involved in migration to the lymph nodes for tumor-derived antigen presentation [29]; therefore, our results suggest that CRM treatment may promote a strong adaptive immune response through the recruitment of APCs that subsequently present tumor antigens to T lymphocytes. Since the low immunogenicity of B16 melanoma model, to define the subpopulations of APC recruited by CRM aerosolization, FVB female mice were i.v. injected with N202.1A cells, an immunogenic murine mammary tumor cell line expressing the rat neu oncogene [14], and treated as described above. A significant increase of CD103+ DCs and plasmacytoid DCs (pDCs) (CD11c+B220+mPDCA-1+ cells) was observed in CRMs versus saline-treated mice, while no difference was detected in conventional DC. Moreover, an increased percentage of alveolar macrophages (AMs) was also observed in this model (Supplementary Figure (available here)). The increase of alveolar macrophages might be related both to an enhanced recruitment induced by autophagic tumor cell-released danger signals and to a reduced apoptosis of these immune cells. Indeed, it has been recently demonstrated that molecules that enhance autophagy make alveolar macrophage more resistant to apoptosis through an attenuation of endoplasmic reticulum and oxidative stress [30].

We then evaluated whether the reduced tumor growth and the increase of APCs were associated with changes in the expression of M1/M2 genes in the lung microenvironment. Suspensions obtained from lung enzymatic digestion were seeded in culture plates to separate adherent cells, which contain macrophages and myeloid cells, from the floating counterpart, mainly constituted by effector cells. Real-time PCR analysis performed on mRNA extracted from the adherent cell fraction revealed a significant upregulation of IL-12, TNF-*α*, and STAT-1 mRNA level, a transcription factor associated with M1 phenotype, in CRM-treated mice, whereas M2-associated genes, such as IL-10, IL-8, and STAT-6, significantly declined (Figure 2(b)). No significant change was detected in IFN-*γ* expression, reported to be mainly produced by T cells after autophagy induction [31]. These findings suggest that aerosolized CRMs are able to shape the immune microenvironment promoting proinflammatory cytokine secretion by macrophage/myeloid cells and reducing the immunosuppression.

The reduction of M2 polarization observed in lung immune infiltrate is in line with the recently published results that demonstrated how intermittent fasting promotes the reduction of TAM polarization and immunosuppressive activity through the inactivation of JAK1/STAT3 pathway in murine models of colon cancer [32].

**Figure 3:**
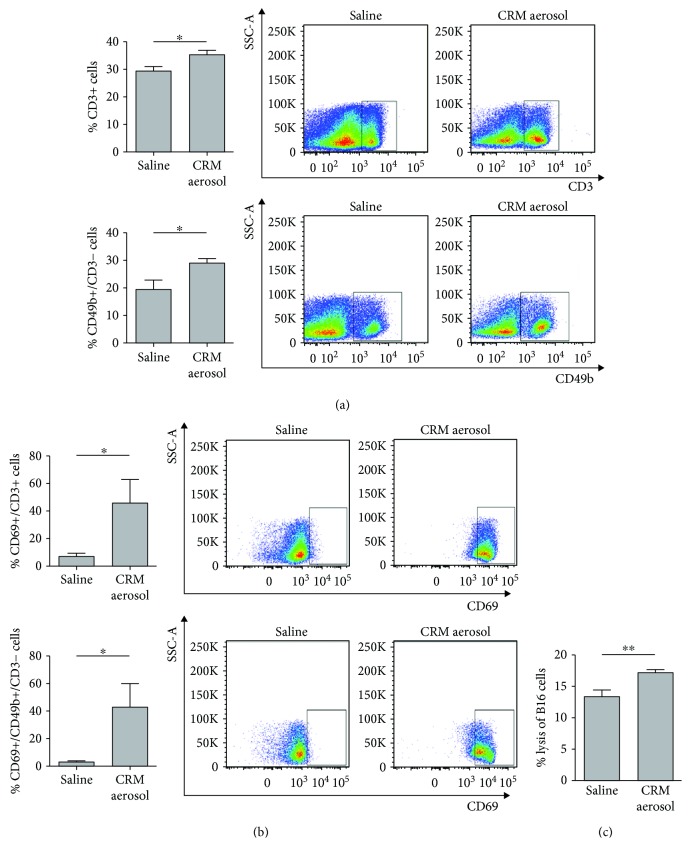
Effects of CRM aerosolization on the recruitment and activation of lung antitumor effector cells. Bars (mean ± SEM) and representative dot plots of the percentage of NK cells (identified as CD45+/CD3-/CD49b+ cells) and T cells (identified as CD45+/CD3+ cells) (a) and of CD69 expressing NK and T cells (b) evaluated in 4 mice/group injected with B16 melanoma cells and aerosolized with saline or combined CRMs. Bars (mean ± SEM) represent the percentage of the specific lysis of B16 target cells cultured for 4 h with nonadherent cells obtained from the lung suspensions of 4 mice/group (c). ^∗^ *p* ≤ 0.05, ^∗∗^ *p* ≤ 0.01 by Student's *t*-test.

We also analyzed the recruitment and the activation status of immune cell population that can directly kill the tumor, NK, and T cells. Flow cytometry analysis of lung immune infiltrate revealed a significant expansion of both NK cells and T lymphocytes (Figure 3(a)) and a significant increase of the expression of CD69 activation marker on the surface membrane of both populations in CRM-treated mice (Figure 3(b)). Moreover, *in vitro* analysis of cytotoxic activity of effector cells contained in nonadherent fractions from CRM-aerosolized mice against B16 melanoma cells revealed a significant increase in the percentage of ^51^Cr-labelled B16 lysis, as compared to control group (Figure 3(b)). Thus, the increase number of APCs and the polarization of the lung microenvironment toward a M1 phenotype promoted the expansion and activation of both T lymphocytes and NK cells. Accordingly, autophagy, besides promoting an adaptive immune response, has been reported to support the maturation of innate effectors, as NK cells [33] both by direct effect on these cells and by the induction of the expression of NK cells activate receptor ligands on cancer cells [34].

In this proof-of-concept study, we investigated the possibility to administer CRMs at the tumor site in the lung of female mice by aerosol, revealing that aerosolized CRMs can reach the bronchoalveolar space and exert antitumor activity. A possible limitation of present study is the use of only female mice, because it is known that constitutive autophagy can be different between male and female due to the biological effect of sex hormones [35, 36], reported to exert extremely different effects, even opposite, in the context of autophagy [36]. However, forcing the cells present in the tumor microenvironment to undergo autophagy by using CRMs, it is possible that we bypassed and flattened the biological effect exerted by sex hormones on autophagy.

In conclusion, this strategy may represent a noninvasive therapeutic approach to locally activate adaptive and innate immunosurveillance, while reducing the toxic effects related to CRM systemic administration.

## Data Availability

The data obtained from FACS and real-time PCR analyses used to support the findings of this study are available from the corresponding author upon request.

## Supplementary Materials

Supplementary Materials Supplementary Figure: modification of APC populations induced by CRM aerosolization in the N202.1A tumor.
