# Supplementary material for: Local Administration of Caloric Restriction Mimetics to Promote the Immune Control of Lung Metastases
Source: J Immunol Res. 2019 Jun 20;2019:2015892. doi: 10.1155/2019/2015892 (PMC6609366; doi:10.1155/2019/2015892)
Supplement: Supplementary Materials — Supplementary Figure: modification of APC populations induced by CRM aerosolization in the N202.1A tumor. [file 2015892.f1.pdf]

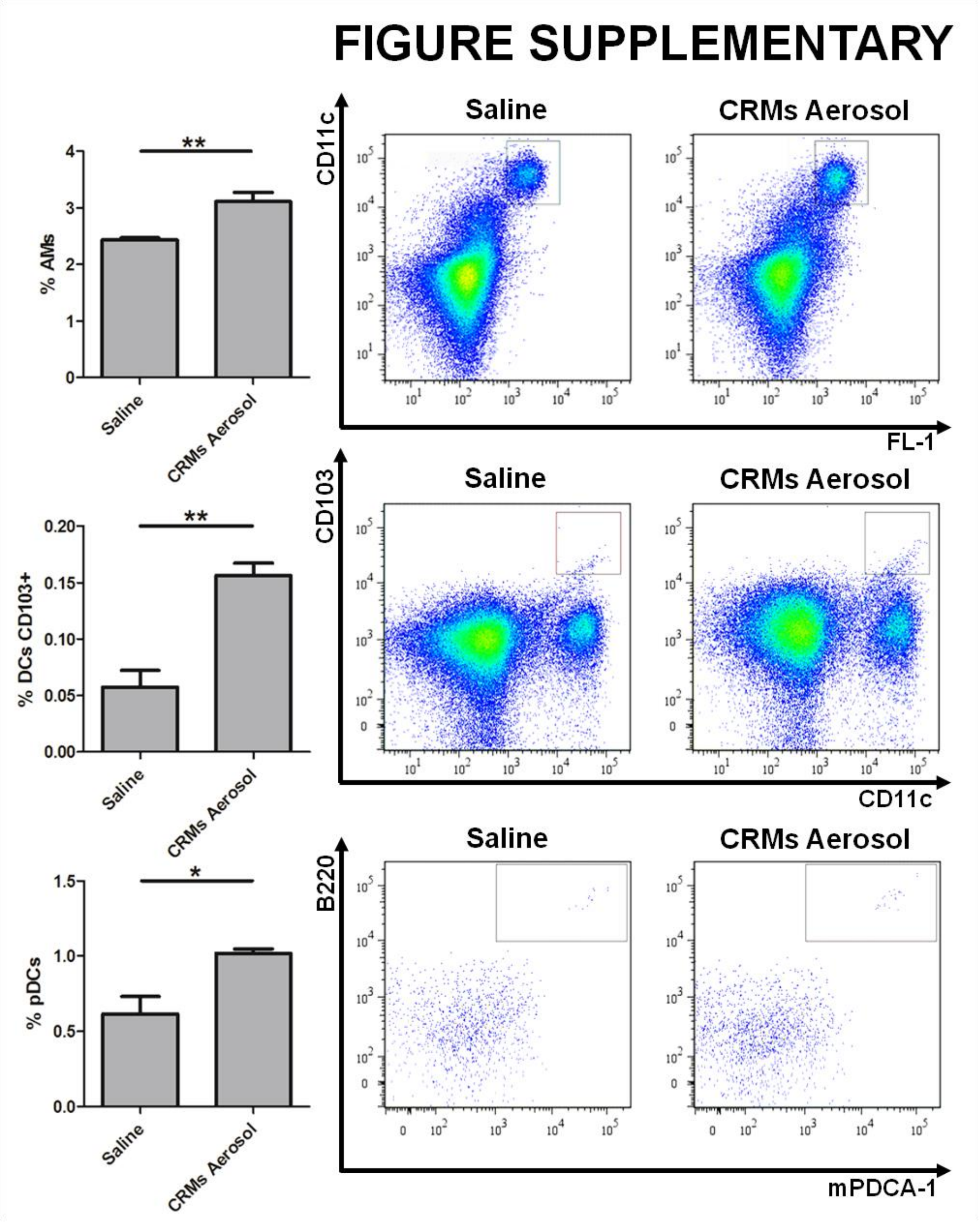

Supplementary Figure. Modification of APCs populations induced by CRMs aerosolization in N202.1A tumor

Bars (mean± SEM) and representative dot plots of the percentage of AMs (identified as CD45+/FL-1+CD11c+ cells), CD103+ DCs (identified as CD45+/CD11b-/CD103+CD11c+ cells) and plasmacytoid DCs (identified as CD45+/CD11c+/B220+mPDCA-1+ cells) evaluated in lung suspensions of 4/5 mice/group injected with N202.1A mammary carcinoma cells and aerosolized with saline or combined CRMs.
